# Induction Heating in Underwater Wet Welding—Thermal Input, Microstructure and Diffusible Hydrogen Content

**DOI:** 10.3390/ma15041417

**Published:** 2022-02-14

**Authors:** Oliver Brätz, Jan Klett, Thomas Wolf, Knuth-Michael Henkel, Hans Jürgen Maier, Thomas Hassel

**Affiliations:** 1 Fraunhofer Institute for Large Structures in Production Engineering IGP, 18059 Rostock, Germany; oliver.braetz@igp.fraunhofer.de (O.B.); knuth.henkel@igp.fraunhofer.de (K.-M.H.); 2 Institut für Werkstoffkunde (Materials Science), Leibniz University Hannover, 30823 Garbsen, Germany; klett@iw.uni-hannover.de (J.K.); maier@iw.uni-hannover.de (H.J.M.); hassel@iw.uni-hannover.de (T.H.)

**Keywords:** hyperbaric wet welding, fine-grained construction steel, preheating, post-weld heat treatment, hydrogen analysis, induction heating, underwater technology

## Abstract

Hydrogen-assisted cracking is a major challenge in underwater wet welding of high-strength steels with a carbon equivalent larger than 0.4 wt%. In dry welding processes, post-weld heat treatment can reduce the hardness in the heat-affected zone while simultaneously lowering the diffusible hydrogen concentration in the weldment. However, common heat treatments known from atmospheric welding under dry conditions are non-applicable in the wet environment. Induction heating could make a difference since the heat is generated directly in the workpiece. In the present study, the thermal input by using a commercial induction heating system under water was characterized first. Then, the effect of an additional induction heating was examined with respect to the resulting microstructure of weldments on structural steels with different strength and composition. Moreover, the diffusible hydrogen content in weld metal was analyzed by the carrier gas hot extraction method. Post-weld induction heating could reduce the diffusible hydrogen content by −34% in 30 m simulated water depth.

## 1. Introduction

Hydrogen embrittlement and hydrogen-assisted cracking (HAC) are known cases of damage, mainly occurring in high-strength steels [1]. The hydrogen-related embrittlement mechanisms leading to material failure are broadly discussed but are not yet clarified in detail [2]. Different theories have been suggested; currently, most scientists agree that a combination of different mechanisms is most probable in many cases [3,4,5]. Despite the ongoing dispute, there is consensus that three basic factors are required for HAC [6,7]:

- a sufficient content of diffusible hydrogen must be present
- a critical local stress state (including residual stresses) is attained and
- a susceptible material or a material in a critical state (microstructure) is used.

All three factors can be found, particularly in underwater wet arc welding [8,9,10,11,12]. During welding, the dissociation of water in the arc atmosphere generates elementary hydrogen, which can easily be absorbed by the weld pool. Water as the surrounding medium leads to high cooling rates after welding. Especially in the heat-affected zone (HAZ), bainitic and martensitic transformations result in microstructures with increased hardness and higher susceptibility for cold cracking. Moreover, wet welding promotes welding distortion and buildup of residual stress. 

In conventional C-Mn steels, the tendency to form brittle HAZ can be predicted based on the carbon equivalent *CE*_IIW_ or *CEV*, Equation (1), proposed by the IIW [13]. Generally, steels with a *CEV* > 0.4 wt% are considered as non-weldable under wet conditions [13].

(1) $$CEV=\frac{Mn}{6}+\frac{Cu+Ni}{15}+\frac{Cr+Mo+V}{5}\;\;(\mathrm{element}\;\mathrm{concentration}\;\mathrm{in}\;\mathrm{wt}\%)$$

Throughout the last 50 years, the scientific community has discussed different solutions for the described challenges. Most of them focus either on the reduction of the diffusible hydrogen content or the optimization of the resulting HAZ microstructure. For instance, promising results were achieved by using new methods, including mechanical support systems for the shielding gas bubble, or ultrasonic-assistance systems in wet flux-cored arc welding (FCAW) [14,15,16,17,18]. Another promising approach is to optimize the composition of the stick electrode’s covering for shielded metal arc welding (SMAW) or the core for FCAW [19,20,21] for wet welding. Changing the microstructure of the weld metal entirely could also bring advantages in terms of reducing the diffusible hydrogen content [22,23,24,25,26]. So far, these methods have mainly been used in laboratory applications only. For the production of high-quality welded high-strength steels joints underwater, studies are being carried out worldwide. Of particular interest is the precise control of heat input in the weld [27,28,29,30]. Currently, for welding of higher strength steels under wet conditions, the only method globally applied by divers is the temper bead welding technique (TBW) [31,32]. This technique implies the additional deposit of functional weld beads on constructional weld beads. These so-called temper beads cause an annealing effect, i.e., reducing hardness and increasing ductility, in the preceding weld bead. This, in turn, leads to a hydrogen soaking effect because the mobility of diffusible hydrogen is enhanced due to the temporary increase in temperature. Furthermore, there is a tempering effect in the HAZ, causing a reduction in hardness. The effectiveness has been studied and proven multiple times in recent past [10,33,34,35]. However, the covering temper bead needs to be removed afterwards, because it possesses an increased susceptibility to HAC. Moreover, this method requires an extremely high skill level and is rather exhausting for the welding diver. In fact, the increased work time and materials costs are substantial compared to welding without TBW. 

Using induction heating technology underwater could be a solution to this issue and was first shown by Zhang et al. [36]. Nevertheless, the methods used were non-applicable concerning practical demands in repair operations. Specifically, the induction heating was used simultaneously during welding. The inductor was located on the backside of the workpiece. However, possible application cases are very rarely found in offshore repair welding. Moreover, a critical reduction of arc stability was found if the inductor was placed too close to the weld region. In other studies, inductive post-weld heat treatment (PWHT) was investigated [37,38]. An improvement of the HAZ microstructure, as well as a reduction of the diffusible hydrogen concentration, was found. However, the induction-source technology used was not safe to be used by divers. This challenge was recently solved by using a different induction system, which was shown to be safely applicable by divers [39,40]. Still, this method needs further investigation for practical application, because heat treatment was not yet characterized for this induction system. Thus, the thermal input of an inductive heat treatment suitable for manual handling underwater was characterized and the resulting microstructure was metallographically investigated in the present study. In addition, numerical simulations of the expected hydrogen concentration within the weld and the resulting diffusible hydrogen content after using the inductive heat treatment were conducted and then validated by thermal desorption analysis.

## 2. Materials and Methods

The induction system used for the experimental investigations was an A4000 by Alesco International AB. The power source operated in the medium frequency range with a fixed frequency of approx. 16 kHz and supplied a maximum power output of 18 kW. A cooled single-turn copper coil with a field amplifier made of a special amorphous metal with high permeability and low electrical conductivity served as the inductor. The inductor was always protected with a PTFE shield resulting in a constant coupling distance of 2 mm. To characterize the thermal input of the induction system, basic studies were performed in a basin filled with 600 L water under constant medium flow. The temperature distribution was measured by using an instrumented steel workpiece sample (plate). In the sample, various thermocouples (type K) were installed with a distance of 10 mm each at material depths from the surface between 1 and 8 mm. The induction head was moved over the workpiece by a mechanized manipulator. The results were compared to temperature measurements made on actual welding samples to investigate any discrepancies due to the geometry of the deposited weld. In this case, the thermocouples were installed into the HAZ of previously wet welded bead-on-plate samples.

Since the induction system worked at a significantly lower frequency compared to prior research, different temperature profiles were expected in the samples [37]. Research regarding PWHT for underwater hydrogen reduction normally works with locally higher temperatures [37] than expected in the present study, or dwell periods are used that are impracticable for manual application by divers [41]. Thus, it was uncertain whether PWHT using the induction system could significantly lower the diffusible hydrogen content in the weld metal at all. Since the variance in hydrogen analysis for wet welding is usually relatively high [42], a large sample number (*n*) is needed to obtain statistically meaningful results. In wet welding, the welding itself, handling, and analyses of large sample batches require lots of effort and time. Welding and analysis of diffusible hydrogen samples were performed in maximum conformity with the ISO 3690 (adopted to wet welding as explained in [43]). Hydrogen analysis was performed applying the carrier gas hot extraction method (CGHE) by thermal desorption at 400 °C for a dwell time of 30 min using a Bruker G4 analyzer.

**Figure 1:**
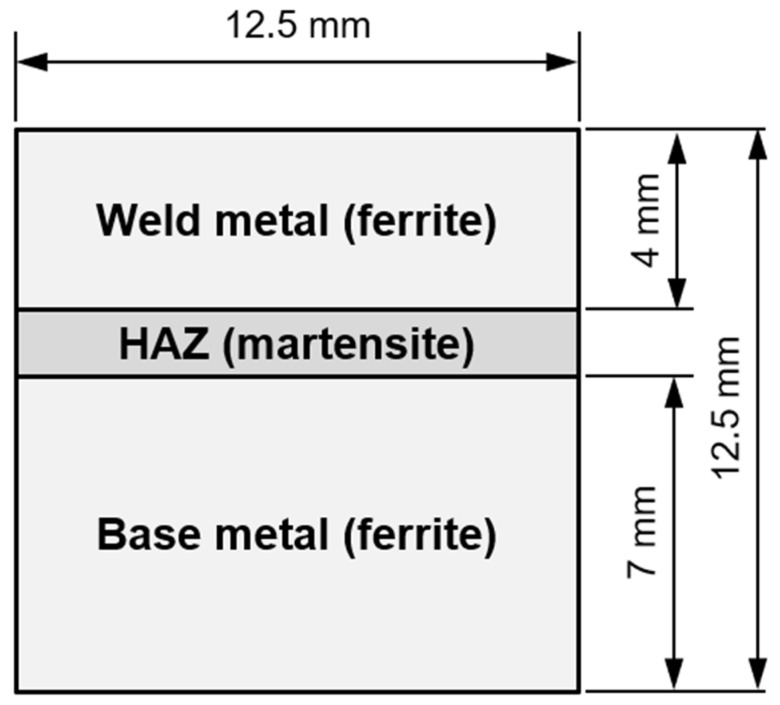
Geometry used for numerical modelling of hydrogen diffusion in the weld; see main text for details.

As it was not known beforehand whether the necessary time-temperature combinations for hydrogen soaking (e.g., [44]) could not be realized with the induction technology in underwater wet welding, a numerical simulation was performed initially. The objective was to obtain an estimate of the hydrogen diffusion profiles within the welds and see whether the inductive PWHT application potentially could result in a significant reduction in diffusible hydrogen concentration in the weld metal by thermal supported diffusion. For the diffusion simulation, a simplified model of a weld bead was used; see Figure 1. The weld metal’s microstructure was assumed to consist predominantly of a ferritic cast structure, whereas the HAZ was modeled as a fully martensitic structure. The initial hydrogen concentration in the weld metal (WM) at the beginning of the calculation was set to *C*_H, WM_ = 1. In other words, the hydrogen is assumed to dissolve rapidly in the weld pool and is evenly distributed there upon solidification. The initial concentration in the HAZ and the base metal (BM) were set as *C*_H, HAZ_ = *C*_H, BM_ = 0, as the weld pool solidifies and cools down rapidly such that hydrogen diffusion is effectively curtailed. The hydrogen concentration at the outer borders was set as *C*_H, Border_ = 0 throughout the simulation. These conditions were used to closely mimic those of prior studies. For the numerical simulation, a 125 × 125 grit size was used and a Crank–Nicolson finite-difference algorithm along with the alternating direction implicit method was employed to solve the diffusion equation, i.e., Fick’s 2nd law, in two dimensions. The temperature-dependent diffusion coefficient in ferrite was calculated, Equation (2), as [8,45,46]

*D*_H_(*T*) = 1.5 × 10^−7^ exp(−0.088 eV/k_B_*T*) in m^2^s^−1^ (2)

where k_B_ is the Boltzmann constant and *T* is the absolute temperature. As the corresponding temperature-dependent diffusion data for martensite are not readily available, it was assumed that, at any given temperature, the effective diffusion coefficient in the martensite is an order of magnitude lower than in the ferrite. This does reflect the fact that martensite possesses a substantially higher trap density for hydrogen. Finally, the temperature-time course in the weld was based on actual data obtained with the induction coil, cf. Section 3.1.

**Figure 2:**
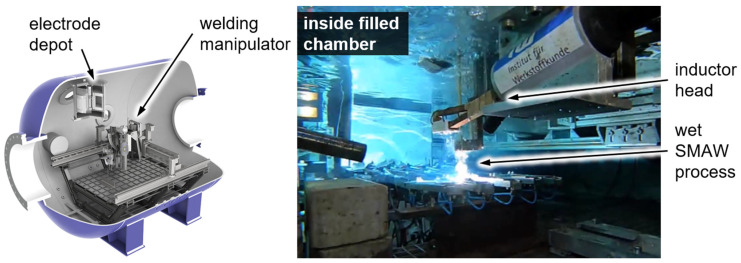
Pressure chamber for mechanized welding at simulated water depths and wet SMAW welding of hydrogen samples with currently uninvolved inductor in rest position.

A pressure chamber was used to experimentally simulate wet welding at different water depths. The chamber holds up to 10 m³ of fresh water and provides a maximum pressure of 30 bar corresponding to a nominal water depth of 300 m. Inside the chamber, there is a welding unit composed of four linear axes, as well as tilting and rotational axes. The welding power source is an ELIN SWA 303 converter supplying up to 300 A. Together with an automated voltage control of the welding unit, reproducible welds can be made in adjustable water depths. This welding unit allows for performing welding in all positions according to ISO 6947. Additionally, a specific clamping system for the preparation of weld samples for diffusible hydrogen analysis based on ISO 3690 was installed into the chamber. The system allowed welding in accordance with all the limitations prescribed by ISO 3690, including the transport of the samples in liquid nitrogen within 20 s for storage just after welding. The induction system was installed into the chamber, as shown in Figure 2. The induction parameters were varied between 70 and 100% of the inductions system’s maximum power. The time used for the PWHT was varied between 20 and 60 s. The induction head was placed directly on top of the weld bead (constant distance of 2 mm achieved via a PTFE cover—Figure 2. The time between welding and storage in liquid nitrogen was held constant at 120 s for all samples. The reference samples were kept in the water within this 120 s, too. For the PWHT samples, the heat treatment was performed within this time and the samples were kept in the water for the rest of the 120 s period. 

For microstructural analysis, samples were sectioned from weldments conducted at four different simulated water depths (*WD*) on different steel grades. Stick electrodes for wet welding of the type Aquaweld by Kjellberg Finsterwalde with a diameter of 3.25 mm complying with DIN 2302—E 38 0 Z RB 2 20 fr (PA, PB, PE, PG) were used as consumables. The welding parameters used at the different water depths are listed in Table 1.

The chemical compositions of the base materials used were analyzed by optical emission spectroscopy. The results are summarized in Table 2. All plates for weld tests have the same thickness of *h* = 10 mm. 

**Figure 3:**
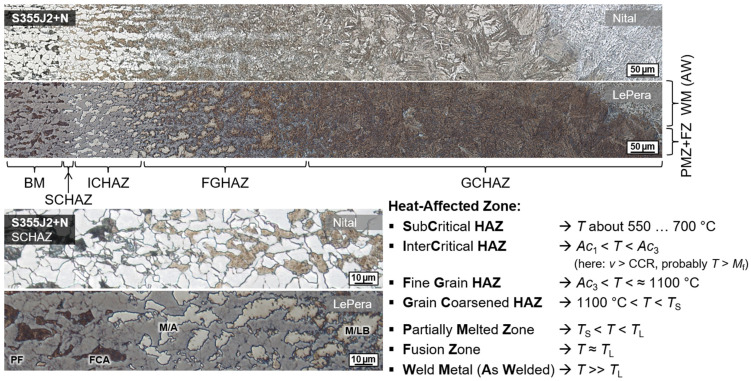
Exemplary HAZ microstructure of wet SMAW weldment on unalloyed structural steel S355J2+N (*WD* = 0.5 m, *h* = 10 mm, *E* = 1.85 kJ/mm).

To evaluate the material effect of induction heating in wet welding of structural steels, the microstructure was analyzed in cross-sections. For a more precise metallographic investigation of the welded region, two preparation techniques were used in a complementary manner to better contrast the microstructure, cf. ref. [47]. A common grain etching with Nital (3% nitric acid in ethanol) was employed to analyze the ferritic matrix. Furthermore, color tint etching according to LePera (4% picric acid in ethanol and 1% sodium metabisulfite in distilled water mixed in 1:1 volume ratio) was used to highlight high-carbon martensite and residual austenite phases (M/A), see Figure 3.

## 3. Results

### 3.1. Under Water Induction Heating

**Figure 4:**
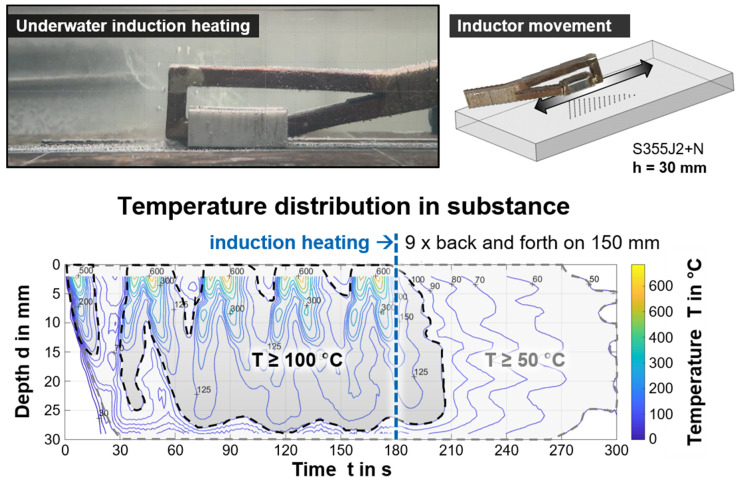
Resulting temperature field in the middle of the longitudinal section of a plate by underwater application of induction heating; *th* = 30 mm, moving inductor, back and forth with 10 mm/s, total duration 180 s.

First, pre-tests were run to determine a suitable heating procedure with the underwater inductive heat input, with a focus on practical, manual use in weld applications. Heat distribution fields describing the temperature as a function of the location, i.e., depth from surface, and time were obtained by instrumented model samples of structural steel S355J+N for various operational parameters and conditions. The temperature transients of different material depths during the run over of the inductor were synchronized and evaluated [40]. Exemplarily, the result of a multi-run back and forth scan for a total duration of 180 s on a plate with 30 mm thickness is shown in Figure 4 as an isotherm diagram. The middle position of an imaginary section along the induction heated length was indicated. The plate heated up with every pass successively through thickness over time (left of the dashed blue line in the diagram). Then, heat dissipation occurred time-dependently. 

These data allowed an application-oriented determination of expected component temperatures for different induction parameters. Due to the underwater specific boundary conditions, the resulting heat distribution fields are more rapidly changing with time compared to conventional welding applications. In the present study, only the maximum induction power level ensured efficient underwater component heating and, further, a moving application was necessary to avoid unintended phase transformations. Based on the data, an underwater application could be carried out manually, as the influence of the inductor feed and slight misalignments of the inductor’s head did not significantly affect the resulting temperature field in the component. Thus, there was only a need of specifying an appropriate induction duration for a given area. However, exceeding 60 s induction duration was of no avail with the induction system used, as the heat was continuously dissipated into the component, such that the temperature level was no longer significantly increased. Depending on the material thickness, two- or three-dimensional heat dissipation must be considered. For thicknesses up to 10 mm, a temperature field that is nearly homogenous in depth over time can be expected. However, it should be noted that the resulting heat distribution in underwater application largely depends on the actual induction parameters, e.g., frequency, inductor geometry, induction power, the environmental conditions (water temperature, flow rate, etc.), the materials properties (heat capacity and conductivity), and the geometry of components, as well as the presence of magnetic field concentration methods. 

### 3.2. Hydrogen Concentration 

**Figure 5:**
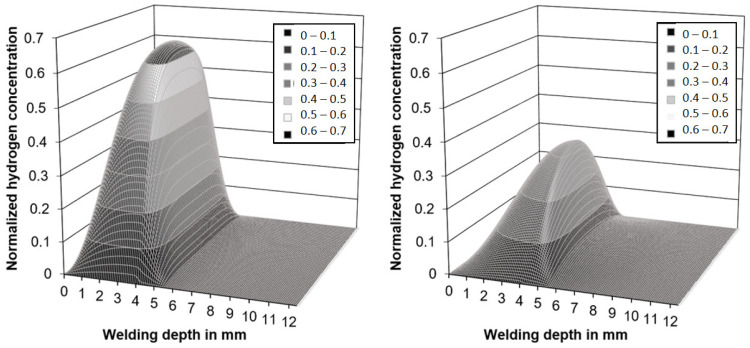
Calculated hydrogen distribution in the simplified weld model, after 300 s without (**left**) and with induction heating (**right**).

Both the measured temperatures and the PWHT time feasible in diving operations were low in comparison to traditional hydrogen soaking operations [41,44]. However, the simulations shown in Figure 5 revealed that a substantial redistribution of hydrogen within the microstructure could be expected upon induction heating despite the short period of 300 s. 

**Figure 6:**
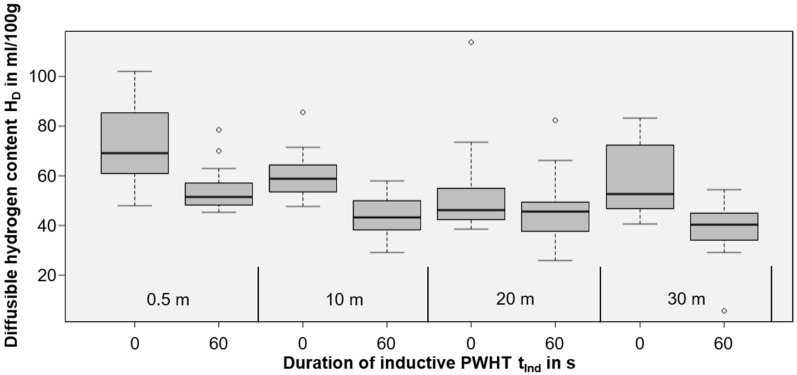
Box plot of the diffusible hydrogen content *H_D_* for welding at different water depths and different induction durations (without PWHT marked as 0 s; 60 s for PWHT).

While the results are not valid in a quantitative way, as the diffusion coefficients are not known for the real microstructures found in wet weldments, the trends justified the experimental effort necessary for measurements of the diffusible hydrogen content in accordance with ISO 3690. As the base metal, unalloyed structural steel of grade EN 10025-2-S235 (*CEV* = 0.31 wt%) was chosen. The parameters used were identical to those shown in Table 1. A total of *n* = 340 samples were welded to ensure statistically meaningful data. The best results were obtained with the maximum power (100%) and 60 s induction duration (*n* = 103 analyzed samples). Tests with lower induction power or lower PWHT times resulted in insufficient influence on hydrogen concentration and were neglected in the following. The results of the corresponding hydrogen measurements are shown in Figure 6.

From Figure 6, the diffusible hydrogen content H_D_ in the deposit weld metal could be reduced for all water depths by using short-time PWHT (60 s, 100% induction power, moved inductor head on 100 mm). Since the variances are large, Welch’s two-sample *t*-tests were calculated for every water depth individually to statistically examine whether there were actual reductions of the diffusible hydrogen content. These test results are listed in Table 3. Establishing an alpha-level of 0.05, the diffusible hydrogen reduction is statistically significant for every water depth except 20 m.

### 3.3. Metallographic Analysis

**Figure 7:**
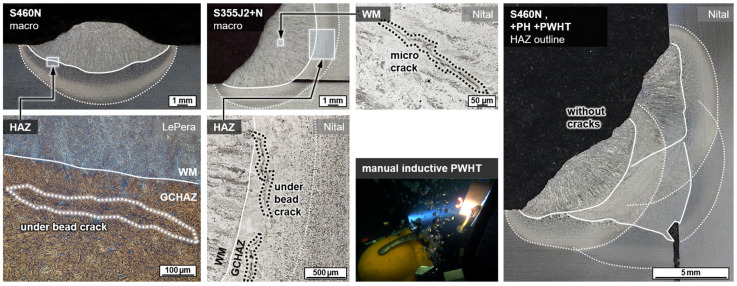
Exemplary occurrence of cold cracks in underwater welds at *WD* = 0.5 m for bead-on-plate (**left**) and fillet weld (**middle**) and positive effect of induction heating in multi-layer wet SMAW of a fillet weld without cracks (**right**).

Wet welding typically results in very high cooling rates due to the convective heat transfer by the surrounding medium. Thus, for C-Mn construction steels, shear-dominated phase transformations were triggered in the weld metal and the HAZ. The result was a typical microstructure with high strength, but low ductility and toughness, cf. Figure 3. These microstructures were particularly susceptible to cold cracking and HAC. Some of the bead-on-plate welds with the unalloyed structural steel grade S355J2+N already showed macroscopic underbead cracks (length > 500 µm) in the grain coarsened heat-affected zone (GCHAZ) parallel to the fusion line. Furthermore, microscopic cold cracks could be detected sporadically in the weld metal, cf. Figure 7. Exemplarily, the positive influence of additional induction heating was demonstrated for a relatively critical application of underwater SMAW fillet welding of S460N. A test piece could be wet welded with three passes without the occurrence of cracks. The induction was applied for pre-heating (PH) as well as PWHT for every run. The induction power was 100% and the induction head was moved manually on a length of about 100 mm for 60 s.

**Figure 8:**
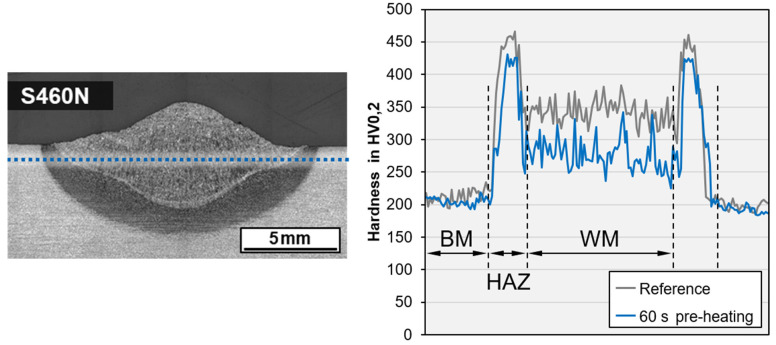
Macro section of wet SMAW on S460N with test indication and comparison of hardness data without induction heating and with PH (*WD* = 0.5 m, *h* = 10 mm, *E* = 1.85 kJ/mm).

A positive influence on the microstructure as a result of inductive preheating could not be verified by light optical microscopy; however, a reduction in the hardness level in the GCHAZ and the weld metal of up to 50 HV could be demonstrated exemplarily for a bead-on-plate weld on the higher-strength steel grade S460N, cf. Figure 8. Here, the length of the deposit weld (100 mm) was preheated (PH) for 60 s with 100% induction power and moving head.

However, in general, inductive preheating does not seem to be feasible in terms of active heat management in contrast to conventional applications. For weldments on S355J2+N and S690QL, no reduction in hardness level was found. As expected, due to its low *CEV*, the steel grade S355MC showed, even under very high cooling rates, no significant hardening in the HAZ, neither without nor with pre-heating. The results of hardness testing in sectioned bead-on-plate welds are listed in Table 4. The measurements were performed as line test in 1 mm distance to the plate surface, c.f. Figure 8. 

**Figure 9:**
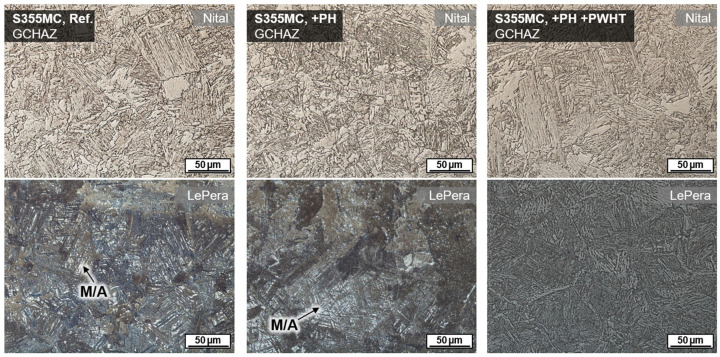
Microstructure in GCHAZ of wet SMAW on S355MC (*WD* = 0.5 m): Reference without induction heating (**left**), with additional PH (**center**), and with combined PH and PWHT (**right**); induction heating each 60 s, 100% induction power, moving induction head on 100 mm length.

The effect of a tempering treatment using inductive PWHT was further validated by investigations using the thermomechanical rolled fine grain steel S355MC. This steel usually shows M/A microphases because of the local segregation of alloy elements in interferritic areas. These microphases could be detected by LePera’s etchant, cf. Figure 9. The usage of induction technology induced a temperature field that was intense and wide enough to even cause annealing transformations in the underbead HAZ region. Thus, M/A was tempered, and no white islands remained in GCHAZ. 

## 4. Discussion

The experiments demonstrated that preheating is assumed to be not efficient for wet welding. Even when the temperature of the components was above 100 °C directly after heating, the cooling of the near-surface region by convection was way too rapid to effectively reduce the cooling rates upon welding. This is in accordance with earlier studies that used different induction systems [37]. Hence, analytic approaches according to [27] for the prediction of preheat temperatures and calculation of cooling times according to EN 10112 or IIW guidance are not applicable to wet welding. Regarding active heat management, only simultaneous heating seems are effective for reducing the cooling rates. However, this approach causes further issues in practical application [36]. The inductive PWHT approach regarding tempering as a substitution for TBW differs from former studies in which the aim was to completely transform the microstructure [37]. With the latter approach, it is necessary to heat above the *Ac*_3_ temperature and to subsequently pass a controlled cooling procedure with the requirement to affect the entire weld region. In the present study, a significant hardness reduction was not obtained with the method applied; however, the tempering effect was verified. In general, the tempering with elevated temperatures caused thermo-activated diffusion, i.e., in metastable martensite and lower bainite (M/LB), carbide segregation occurred. The strength and the corresponding hardness remained at about the same level, but toughness was increased, which is relevant, particularly concerning HAC [6]. 

**Figure 10:**
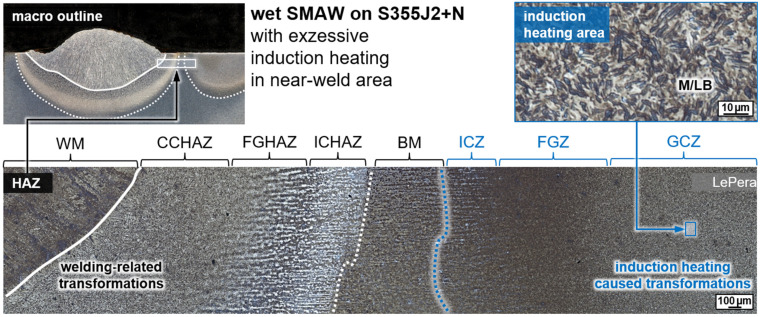
Microstructure in the HAZ of wet SMAW on S355J2+N demonstrating unintended transformation in the near-weld area; see main text for details.

The risk of an unwanted phase transformation in the near-weld seam area because of excessive induction treatment could also be demonstrated metallographically, as in Figure 10. Thus, if transformations into austenite occur during heating, there is a serious risk of obtaining hardened microstructures of martensite and lower bainite (M/LB) due to the quenching effect just upon ending the induction heating. To avoid these unintended transformations, the high power induction heating system should not be used without movement. Instead, other induction equipment that allows for a power-regulated final cooling down process should be applied. However, this approach is technically more extensive because it requires a measurement of the workpiece temperature as a controlled process variable or a power course, which must be qualified individually for the present workpiece geometry [37,38].

Compared to previous work [37], only a small reduction in hardness was observed in the present study. This is reasoned by the approach of applying induction heating for the purpose of tempering with high practical relevance (possible substitution of TBW) instead of normalization with a controlled cooling rate. In addition, the reduction in the hydrogen content in the weld was also less pronounced because of the relatively low soaking times, which are justified by the approach using practical PWHT durations for manual operation in the first instance. Nevertheless, an improvement in weld quality can be achieved with this proven and safe technology. The applicability of this process was verified by divers and was reproducibly applied.

The trends expected based on the diffusion simulations were validated by the experimental investigations. Yet, the actual diffusion coefficients as a function of temperature for a given material with specific microstructural composition are needed to allow for quantitative predictions. Specifically, diffusion coefficients for the weldment, heat-affected zones, and their transition zones are needed; the diffusion data should also include the austenite temperature range to allow considering the effect of phase transformation on the evolution of the local hydrogen concentration. These data are not yet available, and work is underway to address these issues.

## 5. Conclusions

The effectiveness and usability of induction heating for wet shielded metal arc welding applications were validated. The main results can be summarized as follows:

I. Commercial induction technology can be used for heating operations with low effort in an underwater welding application.
II. The inductive heat input is appropriate to perform preheating as well as post-weld heat treatment (PWHT) measures. Yet, attention must be paid to avoid unintended transformations due to a possible quenching effect upon induction heating.
III. Preheating for the purpose of active heat management during welding or to reduce the cooling rate just after welding, is not efficient for wet underwater welding. Only for bead on plate welds on S460N was a hardness reduction of about 30 HV achieved in the HAZ. For the other used steel grades, no significant influence on the resulting hardness level was found.
IV. Inductive PWHT can be applied to obtain tempering effects in quenched regions of the HAZ.
V. Even a relatively short time usage of inductive PWHT can significantly support hydrogen effusion, and, thus, can reduce the risk of HAC. Diffusible hydrogen content could be reduced by 17 to 34%.

## Figures and Tables

**Table 1:** Welding parameters (mean values of measured process parameters).

Water Depth *WD* in m	Polarity	Voltage *U* in V	Current *I* in A	Welding Speed *v* in m/min	Arc Energy *E* in kJ/mm
0.5	DCEN	33.5	185.0	0.2	1.86
10	DCEN	24.4	202.3	0.2	1.48
20	DCEN	26.1	206.7	0.2	1.62
30	DCEN	26.2	203.3	0.2	1.60

**Table 2:** Chemical compositions of materials studied, in wt%.

Material	C	Si	Mn	Cr	Mo	V	Ni	Cu	P+S	*CEV*
EN 10149-2-S355MC	0.082	0.036	1.07	0.020	0.001	0.004	0.013	0.011	0.015	0.27
EN 10025-2-S355J2+N	0.161	0.194	1.49	0.058	0.021	0.001	0.050	0.033	0.021	0.43
EN 10025-3-S460N	0.168	0.442	1.59	0.036	0.006	0.089	0.530	0.164	0.015	0.51
EN 10025-6-S690QL	0.156	0.382	1.32	0.780	0.361	0.052	0.802	0.044	0.019	0.67

**Table 3:** Reduction of hydrogen concentration in wet welded samples with inductive PWHT.

Effect of Induction PWHT (60 s, 100%)	Welded at Simulated Water Depth of			
	0.5 m	10 m	20 m	30 m
mean value of *H*_D_ in ml/100g	54.8	44.1	45.9	38.4
relative reduction	−31%	−28%	−17%	−34%
statistic evaluation by *t*-tests	*n* = 29 *p* < 0.005	*n* = 22 *p* < 0.005	*n* = 28 *p* < 0.120	*n* = 24 *p* < 0.005

**Table 4:** Hardness values in specific regions of the bead-on-plate welds, references and with PH.

Region	Hardness in HV0,2							
	S355MC		S355J2+N		S460N		S690QL	
	Ref.	+PH	Ref.	+PH	Ref.	+PH	Ref.	+PH
base material	180–215		175–215		185–225		290–315	
HAZ	215–240	210–265	260–450	260–440	260–465	285–430	275–455	270–455
weld metal	215–255	210–245	195–320	200–285	305–380	235–340	240–310	255–300

## Data Availability

Not applicable.
